# Increased healthcare utilisation among atopic children in a general practice database: a nested index-control study

**DOI:** 10.3399/bjgpopen18X101349

**Published:** 2018-04-07

**Authors:** David HJ Pols, Mark MJ Nielen, Arthur M Bohnen, Joke C Korevaar, Patrick JE Bindels

**Affiliations:** 1 Lecturer, Department of General Practice, Erasmus MC, University Medical Center Rotterdam, Rotterdam, The Netherlands; 2 Senior Researcher, NIVEL, Netherlands Institute for Health Services Research, Utrecht, The Netherlands; 3 Assistant Professor, Department of General Practice, Erasmus MC, University Medical Center Rotterdam, Rotterdam, The Netherlands; 4 Programme Coordinator, General Practice, NIVEL, Netherlands Institute for Health Services Research, Utrecht, The Netherlands; 5 Professor, Department of General Practice, Erasmus MC, University Medical Center Rotterdam, Rotterdam, The Netherlands

**Keywords:** allergic rhinitis, asthma, atopic eczema, epidemiology, general practice, healthcare utilisation

## Abstract

**Background:**

Atopic eczema, asthma, and allergic rhinitis (AR) create a serious burden on general practice resources.

**Aim:**

To investigate the use of general practice resources (that is, consultation visits, telephone contacts, and home visits) in children with physician-diagnosed atopic disorders (ADs).

**Design & setting:**

In a nested index-control study design, all children (here defined as individuals aged 2–18 years) listed in a representative general practice database were selected in 2014.

**Method:**

Children diagnosed with ADs were matched on age and sex with non-atopic controls within the same practice. For all the different groups, the number and frequency of children contacting the GP were calculated.

**Results:**

Of the children with atopic eczema (*n* = 15 202), 80% consulted the GP in 2014 (controls = 67%). Of the children with asthma (*n* = 7754), 80% consulted the GP (controls = 65%), and for children with AR (*n* = 6710), this was 82% (controls = 66%). Of the children with all three ADs, 91% consulted the GP (controls = 68%). On average, a child with atopic eczema contacted the GP 2.8 times/year (controls = 1.9); for children with asthma, the contact frequency was 3.0 (controls = 1.9); and for AR, 3.2 (controls = 1.9). For children with all three ADs, the contact frequency was 4.3 (controls = 2.0). Consultations related to the ADs investigated only explain a smaller part of the increased healthcare utilisation in atopic children.

**Conclusion:**

Atopic children use more general practice resources compared to non-atopic children, yet frequently for morbidity or other health-related questions not related to one of the ADs.

## How this fits in

Atopic children use significantly more primary healthcare resources compared with non-atopic children. Remarkably, consultations related to specific ADs only explain a smaller part of the increased healthcare utilisation in atopic children. The majority of the excess consultations were therefore related to morbidity not diagnosed as one of the specific ADs.

## Introduction

Atopic eczema, asthma, and AR are among the most common chronic disorders in children.^1,2^ They are often referred to as ‘atopic disorders’ and represent a burden on general practice resources. However, the extent of this burdern is largely unknown. A recent study, based on health surveys, showed that children with atopic eczema, asthma, and AR used more healthcare resources than children without these disorders.^3^ When studying healthcare utilisation in a general practice setting, a diagnosis based on a physician’s assessment, such as that of a GP, provides more realistic results than questionnaire-based diagnoses, and therefore should be preferred.^1^ Previous studies examining healthcare utilisation of atopic children were often conducted in different clinical settings (for example, birth cohorts). Also, whereas most of the studies on healthcare utilisation have focused on asthma,^3–9^ only a few focused on atopic eczema^3,10^ and AR.^3^ All these studies demonstrated that the healthcare utilisation of atopic children is significantly higher compared with non-atopic children. However, to the authors' knowledge, no study has examined to what extent this increased use of healthcare resources reflects extra consultations regarding the ADs, or reflects consultations regarding non-atopic comorbidity (for example, consultations for common symptoms occurring in childhood).

The present study aimed to quantify the current health burden posed by specific ADs (atopic eczema, asthma, and AR), on general practice resources, based on electronic health records. Furthermore, a differentiation is made between atopic-related consultations and non-atopic related consultations.

## Method

### NIVEL primary care database

Generally, all non-institutionalised residents in the Netherlands are registered in a general practice, even if they do not contact the GP. Since 2001, NIVEL-Primary Care Database (NIVEL-PCD) includes routinely extracted data from electronic health records (EHRs) from a representative sample of Dutch general practices,^11^ including information about declared encounters, prescribed medication, and diagnoses. Diagnoses are recorded and classified according to the International Classification of Primary Care 1 (ICPC-1).^12^ In 2014, the authors used data from all NIVEL-PCD practices (with ≥500 listed patients; mean practice size = 2350 patients) with sufficient data quality, fulfilling the following criteria: complete medical and financial registration of encounters (defined as ≥46 weeks per year), and sufficient ICPC-1 coding of diagnostic information (defined as ≥70% of the recorded encounters with an ICPC-1 code). An additional requirement was a minimum follow-up of 3 years for an individual child (that is, data had to be available for 2012–2014), to reduce the risk of registration bias; for this reason, only data for children aged ≥2 years are presented here.

### Selection of atopic children

Based on a method that is described in detail elsewhere,^2^ all children with specific ADs (represented by the ICPC-codes S87: atopic dermatitis, R96: asthma, and R97: AR) were selected on 1 January 2014. In practice, an atopic diagnosis was maintained if (based on available data from EHRs in the period 2002–2014) the child had ≥2 contact moments in that episode of care (for S87; R96; R97), and had received ≥2 relevant prescriptions. In the Dutch setting, prescriptions are linked with a code based on the Anatomical Therapeutic Chemical Classification System (ATC), making the identification of prescriptions possible. For atopic eczema, the ATC code D07 (dermatological corticosteroids) was used; for asthma, the ATC code R03 (drugs for obstructive airway diseases) was used; and for AR, the ATC codes R01AC (nasal preparation of antiallergic agents, excluding corticosteroids), R01AD (nasal preparation of corticosteroids), and R06 (antihistamines for systemic use) were used. These medication proxies have been tested by Mulder *et al* using registered diagnoses as a gold standard.^13^ If the child did not meet these criteria, the child was considered not to have that AD. It was not a requirement that the patient had contacted the GP in 2014 for that specific AD.

### Atopic triad

In contrast to the traditional classification of children with atopic eczema, asthma, or AR, a fourth distinct group of children with all three ADs might exist.^1^ Therefore, ‘atopic triad’ (AT) episodes were developed for research purposes to learn more about this potentially unique group of children. An AT was only defined when a child was diagnosed with all three ADs (corrected to select cases with a higher probability of the clinically-relevant disorder), based on available data from EHRs in the period 2002–2014.

### Design

In a nested index-control study design, one matched control patient (no recorded ICPC-1 codes S87, R96, or R97 ever) within the same general practice was selected for each atopic child, based on sex and age in 2014. When studying children with atopic eczema, asthma, or AR for this study, only those children that had one AD were selected. For children with all three atopic disorders, one matched control patient was also selected.

### Statistical analyses

In the Netherlands, a financial declaration is automatically created in the EHRs at the end of every consultation; that is, consultation visits, telephone contacts, and home visits (the ordering of repeat medication was excluded). Financial declaration recordings from the year 2014 were therefore used to determine healthcare utilisation in general practice. Diagnoses were linked with declared encounters on the same day. If a child consulted the GP for both an atopic-related problem as well as for a non-atopic-related problem, the declared encounter was considered atopic-related. All patients aged 2–18 years were selected. Two different epidemiological markers were calculated: firstly, the percentage of patients consulting the GP in 1 year, as well as the percentage of patients consulting the GP for the specific AD of interest; and secondly, contact frequency, defined as the number of declared encounters overall, as well as the number of declared encounters for a specific AD in 1 year.

For the year 2014, healthcare utilisation and contact frequency rates were calculated for atopic eczema, asthma, AR, and AT in males and females for the age groups 2–6 years, 7–12 years, 13–18 years, and 2–18 years. For the analyses of children with atopic eczema, asthma, or AR, the child was not diagnosed with any of the other ADs. Statistical differences between the groups were tested using χ^2 ^tests (the percentage of patients consulting) and *t*-tests (contact frequency). Due to multiple testing, differences were considered statistically significant with *P*<0.001. All analyses were performed with Stata (version 14.1).

## Results

### General characteristics

In 2014, 409 312 children were identified from the NIVEL-PCD. From this group, children were identified fulfilling the selection criteria with: only eczema (*n* = 15 202); only asthma (*n* = 7754); only AR (*n* = 6710); and AT (*n* = 555). One control patient was matched for each of these atopic children. For this study, 307 different general practices were involved. With the exception of atopic eczema (48.2%), the majority of the children with AD were male: asthma (58.9%), AR (57.9%), and AT (61.6%).

In both the atopic and non-atopic group, females visited the GP more often compared with males. When examining age in more detail, males showed an overall decrease in consultation rates as they became older, whereas females showed a dip in the consultation rate just before adolescence (7–12 years). Both these trends were the same in atopic as well as non-atopic children (Tables 1 and 2).

**Table 1.** Healthcare utilisation in 2014 for children with single AD (only atopic eczema, only asthma, or only AR) versus controls

	Total children, *n*	Children consulting a GP, %^a^		Children consulting a GP for disorder, %	Contact frequency, contact/year^a^		Contact frequency for disorder, contact/year
		No AD	AD	AD	No AD	AD	AD
**Atopic eczema**							
Male, total	14 662	66	78	22	1.8	2.6	0.3
Male, 2–6 years	6264	72	84	24	2.1	3.0	0.4
Male, 7–12 years	5322	63	75	21	1.6	2.3	0.3
Male, 13–18 years	3076	60	73	23	1.5	2.2	0.3
Female, total	15 742	68	81	26	2.1	3.0	0.4
Female, 2–6 years	5728	71	82	27	2.1	3.0	0.4
Female, 7–12 years	6126	62	77	23	1.7	2.5	0.3
Female, 13–18 years	3888	72	85	31	2.5	3.6	0.5
Total group	30 404	67	80	24	1.9	2.8	0.4
**Asthma**							
Male, total	9132	62	78	27	1.6	2.7	0.5
Male, 2–6 years	2174	72	86	32	2.1	3.4	0.6
Male, 7–12 years	3698	60	78	28	1.4	2.5	0.5
Male, 13–18 years	3260	59	73	23	1.5	2.3	0.4
Female, total	6376	69	83	30	2.2	3.5	0.6
Female, 2–6 years	1440	73	86	35	2.3	3.5	0.7
Female, 7–12 years	2292	63	79	24	1.7	2.7	0.4
Female, 13–18 years	2644	73	85	32	2.5	4.2	0.7
Total group	15 508	65	80	28	1.9	3.0	0.5
**AR**							
Male	7766	62	79	35	1.6	2.7	0.5
Male, 2–6 years	326	75	94	52	2.3	4.4	1.0
Male, 7–12 years	2682	64	82	42	1.6	2.8	0.7
Male, 13–18 years	4758	59	77	30	1.5	2.4	0.4
Female, total	5654	71	87	39	2.4	3.8	0.6
Female, 2–6 years	218	77	95	48	2.2	5.0	0.9
Female, 7–12 years	1608	63	82	41	1.7	3.0	0.7
Female, 13–18 years	3828	74	88	38	2.7	4.1	0.6
Total group	13 420	66	82	37	1.9	3.2	0.6

^a^ *P*<0.001. AD = atopic disorder. AR = allergic rhinitis.

**Table 2.** Healthcare utilisation in 2014 for children with AT

	Total children, *n*	Children consulting a GP, %^a^		Children consulting a GP for eczema, %	Children consulting a GP for asthma, %	Children consulting a GP for AR, %	Contact frequency, contact/year^a^		Contact frequency for eczema, contact/year	Contact frequency for asthma, contact/year	Contact frequency for AR, contact/year
		No AT	AT	AT	AT	AT	No AT	AT	AT	AT	AT
**Male, total**	684	65	89	29	35	35	1.7	3.9	0.5	0.7	0.5
Male, 2–6 years	98	67	98	35	49	53	1.9	5.8	0.5	0.9	1.1
Male, 7–12 years	352	66	89	27	35	32	1.6	3.6	0.5	0.6	0.5
Male, 13–18 years	234	63	85	28	31	32	1.6	3.4	0.4	0.6	0.4
**Female, total**	426	72	93	38	39	40	2.4	5.0	0.6	0.9	0.6
Female, 2–6 years	36	72	94	67	50	44	2.8	5.0	1.2	1.3	0.8
Female, 7–12 years	166	70	94	31	37	39	1.7	4.3	0.5	0.7	0.6
Female, 13–18 years	224	74	93	38	39	40	3.0	5.5	0.7	0.9	0.6
**Total group**	1110	68	91	32	37	37	2.0	4.3	0.5	0.7	0.6

^a^ *P*<0.001. AR = allergic rhinitis. AT = atopic triad.

### Children with only atopic eczema

In 2014, 80% of the children diagnosed with only atopic eczema consulted their GP, compared with 67% in the control group (*P*<0.001). Of the children with atopic eczema, only 24% consulted their GP because of their atopic eczema. When examining the contact frequency, children with atopic eczema consulted their GP on average 2.8 times/year, compared with 1.9 times/year in the control group (difference 0.9 times/year, *P*<0.001). The average contact frequency for atopic eczema-related consultations was only 0.4 times/year; therefore, 0.5 of the additional consultations in a year were due to other morbidity or other health-related questions. The differences in contact frequencies are not explained by the few children who consulted their GP very often.

### Children with only asthma

In 2014, 80% of the children with only asthma consulted their GP, compared with 65% in the control group (*P*<0.001). Only 28% of the asthmatic children had asthma-related consultations with their GP. Asthmatic children consulted their GP on average 3.0 times/year, compared to 1.9 times/year in the control group (difference 1.1 times/year, *P*<0.001). Since an asthmatic child consulted their GP for asthma-related problems only 0.5 times/year, this implies that an asthmatic child consults the GP 0.6 times/year extra for other morbidity.

### Children with only AR

In 2014, 82% of the children diagnosed with only AR consulted their GP (controls = 66%, *P*<0.001). Of the children with only AR, 37% consulted their GP because of this condition. Contact frequency of children with AR was on average 3.2 times/year (control = 1.9 times/year; difference = 1.3 times/year; *P*<0.001). This difference is 0.6 times/year due to AR, and 0.7 times/year due to other reasons.

### Children with AT

In 2014, only a small group of children were identified as being diagnosed with AT, of which 91% consulted their GP (controls = 68%*, P*<0.001): 32% of these children consulted their GP in 2014 for atopic eczema, 37% for asthma, and 37% for AR. The contact frequency of children with AT was on average 4.3 times/year, compared with 2.0 times/year in the control group (*P*<0.001). The contact frequency for atopic eczema-related consultations was 0.5 times/year, for asthma-related consultations 0.7, and for AR 0.6. Therefore, of the excess consultation rate of 2.3 times/year, 1.8 is caused by AT, and 0.5 is due to other reasons.

## Discussion

### Summary

This study is the first to examine healthcare utilisation of all three specific ADs in a general practice setting, using physician-based diagnoses. This study contributes new and detailed data on the increased healthcare utilisation associated with atopic eczema, asthma, and AR in a sample of Dutch children selected from a representative general practice database. Children with ADs use more general practice resources compared with children without ADs. Remarkably, the excess consultation rates are mainly due to non-atopic symptoms and diagnoses (that is, those symptoms and diagnoses not labelled as any of the studied ADs). In children with AT, a comparable excess rate (0.5 times/year) is caused by this non-atopic morbidity, suggesting that excess morbidity occurred in all four groups at an equal frequency. Nevertheless, children with AT consulted the GP most frequently, indicating that this might be a unique group. ADs did not explain the trends regarding age and sex that were observed in the present study.

### Strengths and limitations

The present study used an extensive and representative primary care database; the number of included children gives this study substantial power. Data from databases are generally considered reliable and there is no risk of recall bias. Furthermore, the present study included only practices with complete data regarding declared consultations. Using physician-based diagnosis of ADs and selecting cases with a higher probability of a clinically-relevant disorder (≥2 consultations and ≥2 relevant prescriptions) made this study highly relevant for studying healthcare utilisation in the general practice setting.

In the Dutch healthcare system, a payment for review for asthmatic children does not yet exist. Most, if not all, consultations in this healthcare system will be ‘family-initiated’. These results might therefore be more applicable to countries which also do not have reviews as part of a quality outcome framework.

Some limitations also need to be discussed. The present study is based on the assumption that the relevant ICPC-1 codes are not missed; however, this risk cannot be excluded, nor can it be quantified. This study also lacks an objective measure of ADs, such as lung function or allergy tests and the results of simple questionnaires to measure the severity of the disorder. For both index patients and controls, the lack of these details could mean that the results were not corrected for an important confounder. The study might also have included some children not currently affected, possibly due to insufficient follow-up by the GP. Finally, although these findings support the hypothesis that childhood ADs increase healthcare utilisation, the precise comorbidity causing the increased healthcare utilisation was not examined.

### Comparison with existing literature

These findings are in agreement with other studies^3–10^ that also concluded that atopic children utilised more health care; however, this study extended those findings by examining whether the extra consultations are a result of a child’s specific AD or are due to other symptoms or diseases. Based on the present study, <50% of the extra consultations can be explained by atopic eczema, asthma, and AR-related consultations. Therefore, the remainder of the consultations is attributed to other symptoms or diseases. Although part of these consultations could still be related to atopy (for example, food allergy or symptoms of undiagnosed ADs), non-atopic-related morbidity will most likely explain an important part of it. It could also be a reflection of parental concern. Future research might further unravel the precise reasons for the increased healthcare utilisation in primary care; could possibly look at the overall healthcare burden; and, preferably, could compare ADs with other chronic conditions in children.

In 2015, a Dutch child (aged 5–17 years) consulted the GP twice a year on average,^14^ which is in accordance with the contact frequency of the control groups in the present study and endorses the conclusions that atopic children utilise more health care due to their atopic constitution. In contrast, older individuals (aged >85 years) had 13 consultations a year.^14^ Unfortunately, it is not possible to compare the healthcare utilisation of atopic children — who are treated mainly by GPs; only a small number of children will be treated exclusively in secondary care — with other chronic conditions in paediatric patients. Diabetes mellitus (DM), autoimmune disorders, and other serious chronic diseases in children are treated mainly by specialist physicians, such as paediatricians, since the prevalence rates of these diseases are too low for GPs to gain the necessary experience. Therefore, problems associated with these chronic conditions in children will most likely be handled in secondary health care. Healthcare utilisation of children with these chronic conditions in general practice can therefore not be compared with ADs, which are mostly treated by GPs. However, when comparing healthcare utilisation of children with ADs with that of adult patients with chronic obstructive pulmonary disease (COPD) and DM, an interesting difference emerges. Of the atopic children, ≥24–37% consulted their GP once a year for their specific AD. This is substantially lower compared to the 54% of patients with COPD who consult their GP for COPD-related problems ≥1 times/year,^15^ or even the 85% of patients with DM that consults the GP ≥1 times/year for this disease.^16^ The most likely explanations for this observation is that, in the Netherlands, adult patients with COPD and DM receive routine follow-up consultations as a result of ‘integrated multidisciplinary care’. Unfortunately, such a follow-up system is not implemented for paediatric patients in general practice. However, identifying patients with asthma with insufficient follow-up and improving their medication management in accordance with asthma clinical guidelines is likely to result in lower healthcare utilisation,^5^ and may improve the quality of life of these children. The Dutch asthma guideline for children recommends ≥1 evaluation/year. ^17^ As shown by others,^18,19^ unawareness and undertreatment of asthma and AR is common, and needs to be addressed. The problem of undertreatment becomes even more relevant when considering, for example, that when AR is undertreated, this can have a negative impact on asthma control.^20,21^

### Implications for practice

Since the contact frequency for ADs is always <1 times/year, there is evidence that atopic children do not receive routine follow-up. Most likely, the majority of these consultations will have been family-initiated, since it is less likely that a Dutch GP initiates consultations. Considering the evidence of both overdiagnosis and underdiagnosis of ADs,^22,23^ the authors suggest that atopic children could probably benefit from better follow-up, for example, as part of integrated multidisciplinary care, and thereby be provided with the care they deserve. Therefore, GPs are urged to be more aware of their atopic paediatric patients and take appropriate action so that they can also benefit from routine follow-up.

In conclusion, atopic children use significantly more primary healthcare resources compared with non-atopic children. Remarkably, consultations related to ADs only explain a smaller part of the increased healthcare utilisation in atopic children. The majority of the excess consultations were therefore related to morbidity not diagnosed as one of the studied ADs.
